# When Hard Doesn’t Mean Malignant: A Benign Axillary Finding in Lobular Carcinoma

**DOI:** 10.7759/cureus.107310

**Published:** 2026-04-18

**Authors:** Abigayle Wyer, Mena Louis, Samuel Berding, Virginia Jarvis, Priscilla Strom

**Affiliations:** 1 Surgery, Northeast Georgia Medical Center Gainesville, Gainesville, USA; 2 Minimally Invasive Surgery, Johns Hopkins University, Washington, DC, USA; 3 General Surgery Residency, Northeast Georgia Medical Center Gainesville, Gainesville, USA; 4 General Surgery, Northeast Georgia Medical Center Gainesville, Gainesville, USA

**Keywords:** axillary lymph node dissection, breast cancer, cdk4/6 inhibitor, heterotopic ossification, invasive lobular carcinoma, osseous metaplasia, sentinel node biopsy

## Abstract

A 50-year-old premenopausal woman presented with an abnormal screening mammogram, which led to the diagnosis of grade 2 invasive lobular carcinoma of the left breast with axillary lymph node metastases. Immunohistochemistry confirmed lobular origin with estrogen receptor (ER)/progesterone receptor (PR) positivity and Human Epidermal Growth Factor Receptor 2 (HER2) negativity. Neoadjuvant chemotherapy with cyclophosphamide and docetaxel was followed by a sentinel lymph node biopsy, which revealed three metastatic nodes. A modified radical mastectomy with axillary dissection was subsequently performed. Postoperative pathology showed residual invasive lobular carcinoma measuring 1.7 cm, with negative surgical margins and angiolymphatic invasion. Notably, a hard axillary mass excised during dissection revealed a rare benign chondroid and osseous metaplastic reaction. This histologic finding, although non-malignant, may be confused with nodal disease intraoperatively or on imaging, underscoring the importance of thorough pathologic analysis. The patient completed post-mastectomy radiation therapy to the chest wall and regional lymphatics and was started on ovarian suppression and endocrine therapy with a Cyclin-dependent Kinase (CDK)4/6 inhibitor. Genetic testing was negative, and there was no family history of breast or ovarian cancer. This case draws attention to an uncommon histopathological response that may follow trauma or biopsy in breast cancer patients and reinforces the need for clinical awareness when interpreting persistent axillary findings post-treatment.

## Introduction

Invasive lobular carcinoma (ILC) represents the second most common histologic subtype of breast cancer, accounting for approximately 10-15% of cases [1]. It is more frequently diagnosed in women over the age of 50 and is typically hormone receptor-positive and HER2-negative [2]. Unlike invasive ductal carcinoma (IDC), ILC tends to grow in a single-file pattern and lacks a strong desmoplastic response, making it more difficult to detect on clinical examination and conventional imaging [3]. Its propensity for multifocality and bilaterality further complicates radiographic assessment, contributing to delays in diagnosis or underestimation of tumor extent [4].

Diagnosis of ILC relies on a combination of imaging, core needle biopsy, and immunohistochemistry [5]. Mammography may miss lobular lesions due to their diffuse architecture, while magnetic resonance imaging (MRI) provides improved sensitivity, especially in dense breast tissue [6]. Histologically, the absence of E-cadherin expression confirms the lobular origin, with additional immunohistochemical markers like GATA-binding protein 3 (GATA3) and Gross Cystic Disease Fluid Protein (GCDFP) supporting a breast primary [7]. In patients with axillary involvement, core biopsy of suspicious lymph nodes offers essential staging information [8]. Increasingly, genomic profiling and receptor status guide treatment selection, allowing for tailored neoadjuvant or adjuvant strategies [9].

Neoadjuvant chemotherapy is often administered in node-positive, hormone receptor-positive breast cancer to assess chemosensitivity and potentially downstage axillary disease [10]. Surgical management following systemic therapy typically involves lumpectomy or mastectomy, accompanied by sentinel lymph node biopsy (SLNB) or axillary dissection, depending on nodal response [11]. SLNB can be technically challenging in patients who have received chemotherapy, as treatment may disrupt lymphatic drainage or obscure nodal mapping [12]. In such cases, pre-treatment clip placement and alternative localization techniques such as Savi Scout markers help identify targeted nodes during surgery [13].

Histopathologic evaluation remains the cornerstone of treatment planning and surveillance [7]. Occasionally, surgical specimens may reveal unexpected findings, such as heterotopic ossification or metaplastic changes [14]. Chondroid or osseous metaplasia is rarely encountered in axillary dissections and may simulate malignancy intraoperatively or on imaging [15]. These benign reactive processes are often associated with prior biopsy, trauma, or inflammation [14]. Awareness of such findings is essential to prevent overinterpretation and ensure accurate staging and appropriate treatment decisions.

## Case presentation

**Figure 1:**
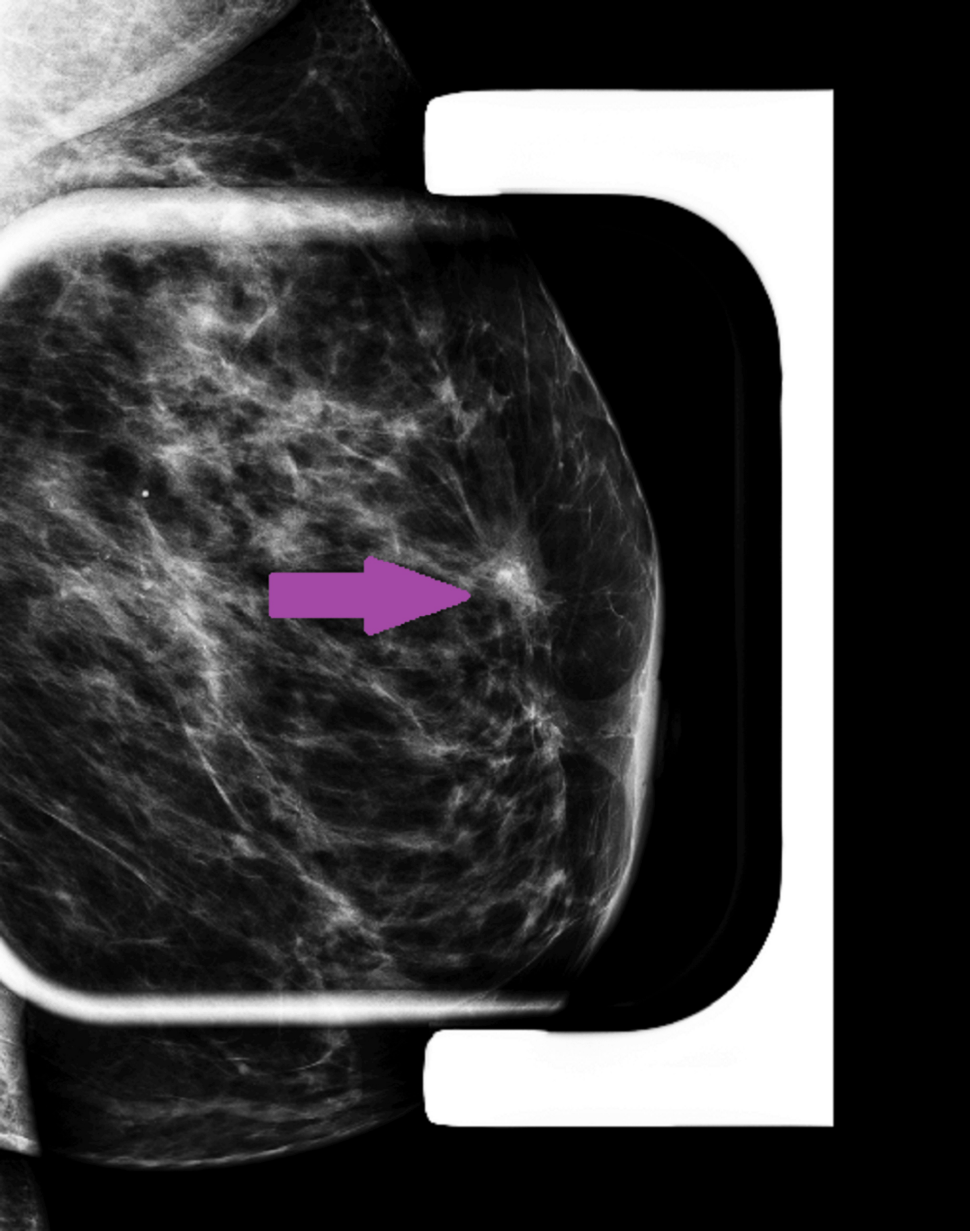
Diagnostic mammogram demonstrates a solid irregular and angulated hypoechoic mass with distortion of surrounding tissue (purple arrow).

**Figure 2:**
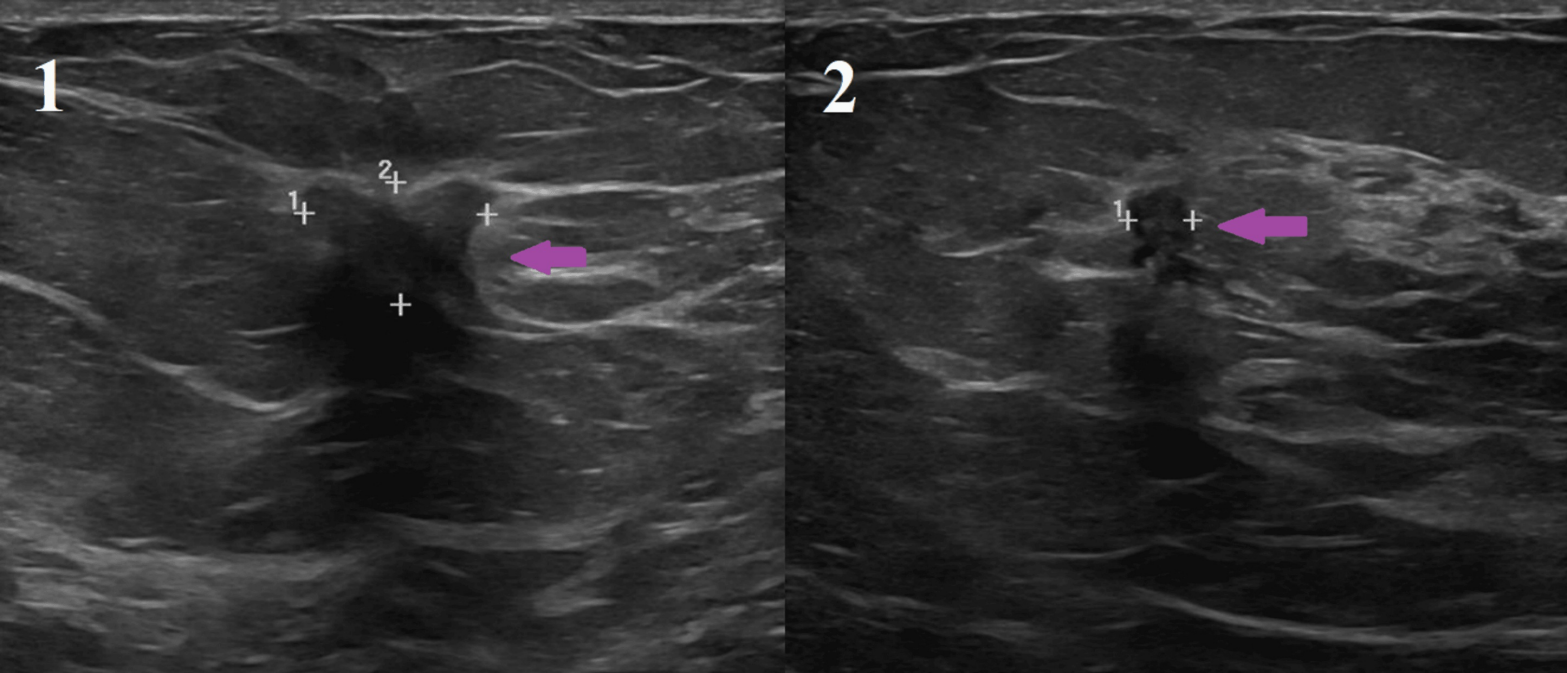
Ultrasound images: Pane 1: a solid irregular and angulated hypoechoic mass (purple arrow) with distortion of surrounding tissue with posterior shadowing at 1 o’clock 2-3 cm from the nipple; Pane 2: a solid hypoechoic mass with irregular angulated margins (purple arrow) with abnormal blood flow at 12 o’clock 2 cm from the nipple.

**Figure 3:**
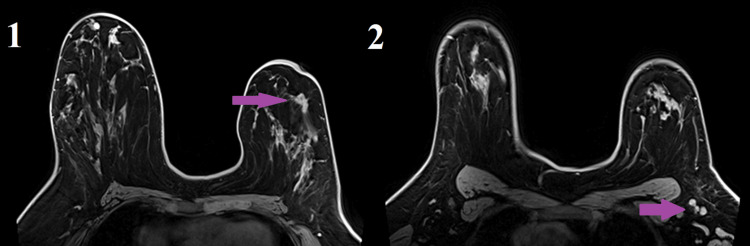
MRI in axial view: Pane 1: an irregular spiculated enhancing mass (purple arrow) at 2-3 o’clock 2 cm from the nipple; Pane 2: abnormal axillary lymph nodes with suspected cortical thickening suspicious for nodal metastasis (purple arrow).

**Figure 4:**
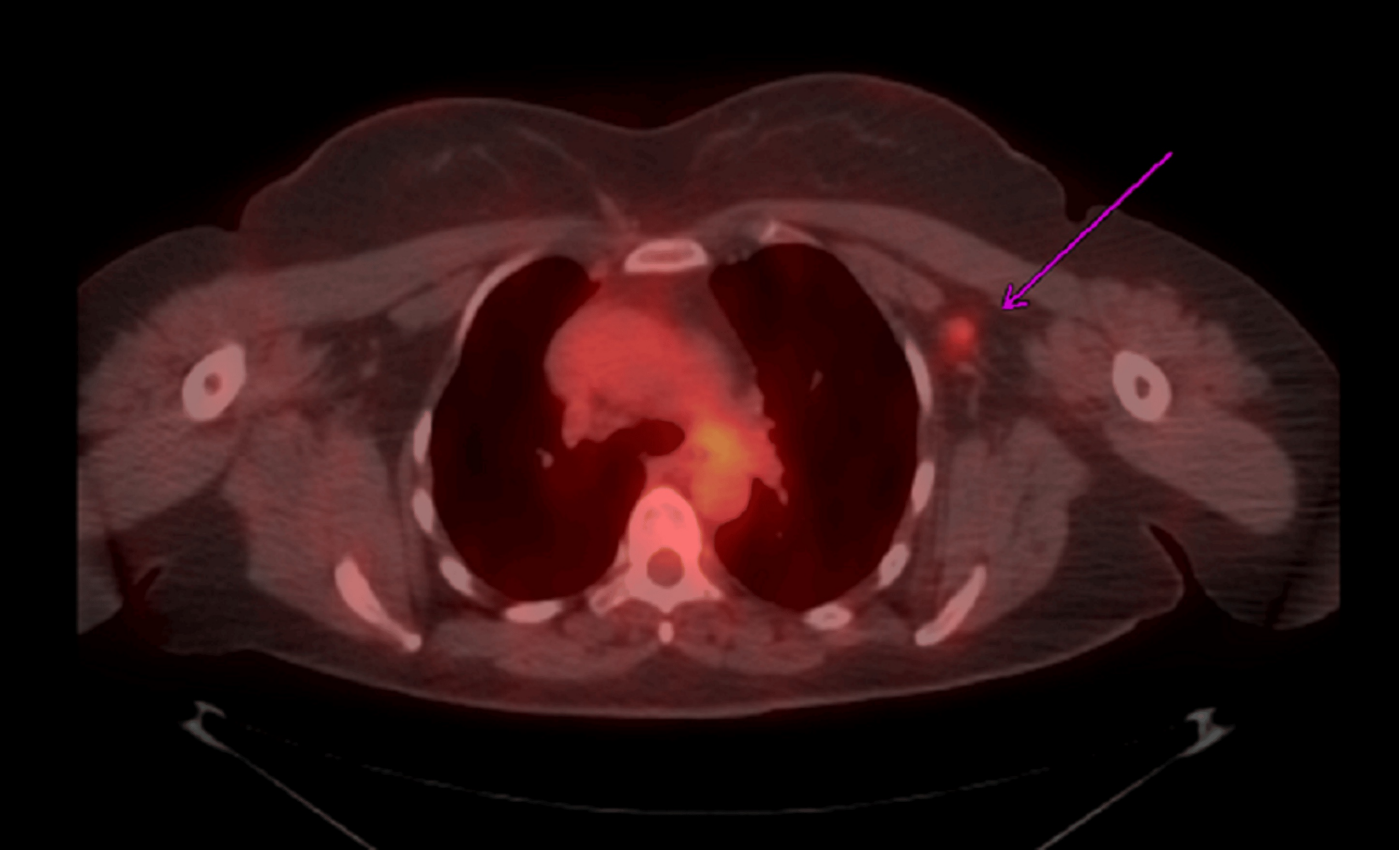
PET scan in axial view demonstrating prominent lymph nodes in the left axillary region with metabolic activity suspicious for metastasis (purple arrow).

A 50-year-old premenopausal woman with no family history of breast or ovarian cancer presented in May 2024 after an abnormal screening mammogram revealed multiple suspicious lesions in the left breast. A diagnostic mammogram identified multiple masses with spiculated margins in the left breast between 12 and 1 o'clock (Figure 1). Diagnostic ultrasound identified a 1.4 cm irregular, hypoechoic mass with posterior shadowing at 1 o’clock (Figure 2, pane 1), a smaller 4 mm irregular mass at 12 o’clock with increased vascularity (Figure 2, pane 2), and a 7 mm cystic lesion also at 12 o’clock. Multiple axillary lymph nodes were noted, the largest measuring 1.3 cm. MRI confirmed a 2.0 × 1.0 × 1.5 cm spiculated, enhancing mass at the 2-3 o’clock position (Figure 3, pane 1), located 2 cm from the nipple, along with multiple additional foci of enhancement without a definite mass and abnormal lymph nodes in the left axilla demonstrating cortical thickening (Figure 3, pane 2). A positron emission tomography (PET) scan in June 2024 showed prominent activity in the left axilla (Figure 4) and a small 5 mm nodule in the right lung.

Core needle biopsy of the left breast lesion confirmed grade 2 invasive lobular carcinoma. Immunohistochemistry revealed estrogen receptor positivity (76%), progesterone receptor positivity (70%), HER2 negativity (1+), and a Ki-67 proliferation index of 35%. Biopsy of an enlarged left axillary node demonstrated metastatic adenocarcinoma consistent with breast origin. The tumor was E-cadherin negative and pancytokeratin positive, confirming lobular subtype, with additional positivity for GATA-3, mammaglobin, and GCDFP. The patient’s follicle-stimulating hormone (FSH) and luteinizing hormone (LH) levels were consistent with premenopausal status. Genetic testing for hereditary cancer syndromes was negative.

She received neoadjuvant chemotherapy consisting of four cycles of cyclophosphamide and docetaxel, administered every three weeks. A Savi Scout reflector was placed in the clipped metastatic axillary lymph node prior to surgery. On October 7, 2024, the patient underwent a left sentinel lymph node biopsy. Intraoperatively, no blue or radioactive nodes were identified. However, the marked lymph node containing the Savi Scout and one firm, palpable node were excised. Pathology revealed metastatic carcinoma in three lymph nodes, with foci measuring 10 mm and 8 mm. There was no extranodal extension.

**Figure 5:**
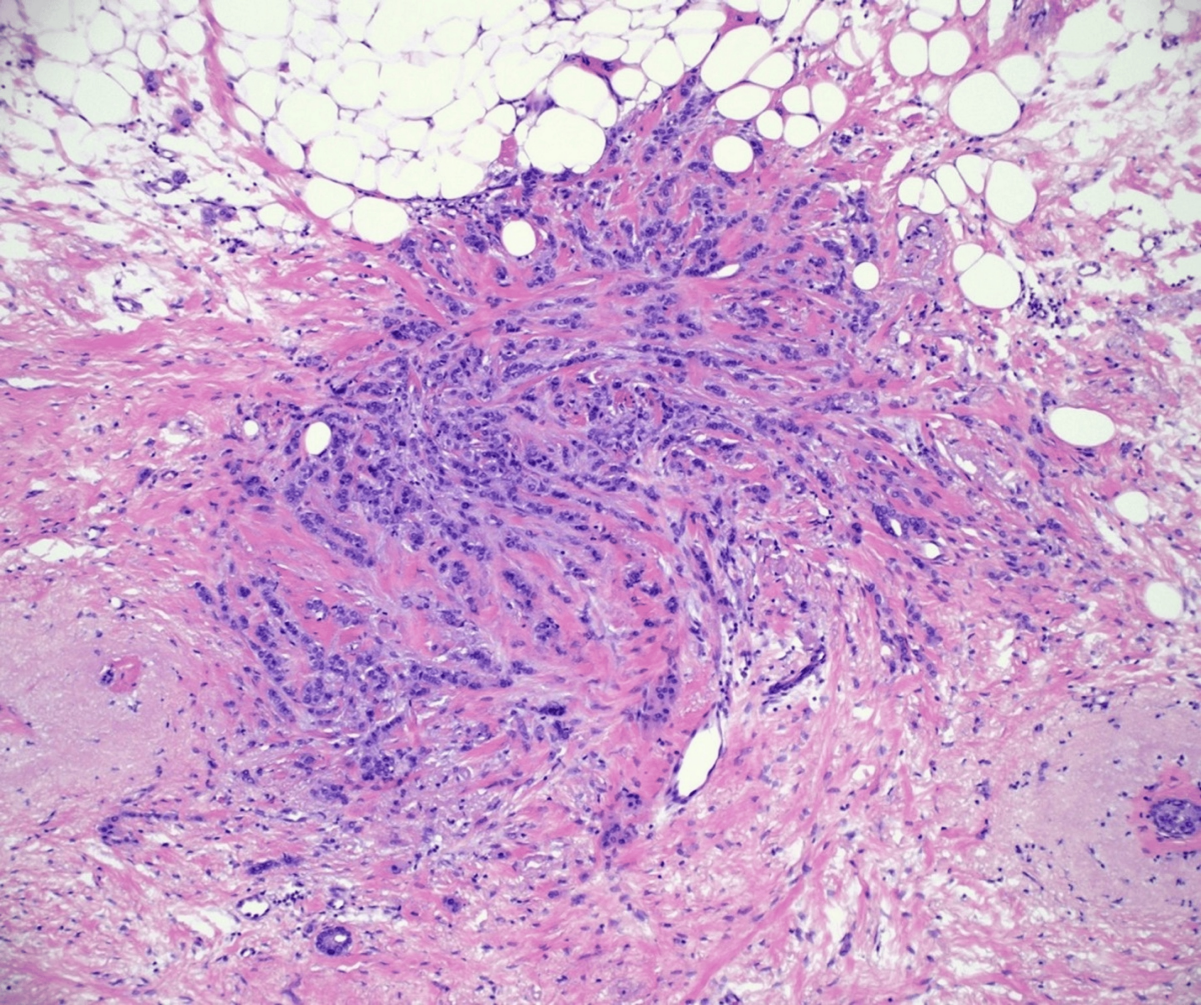
Pathology of invasive lobular carcinoma with gram stain at 4x magnification demonstrates focus of invasive carcinoma and associated fibroelastosis.

**Figure 6:**
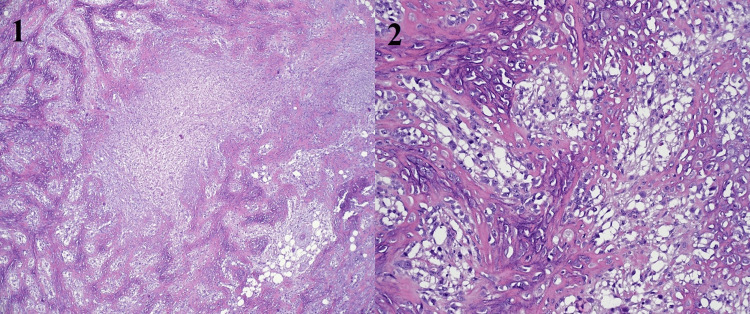
Pathology of axillary tissue with Gram stain demonstrates axillary benign chondroid metaplasia with osteoid at 4x magnification (Pane 1) and 10x magnification (Pane 2). No malignancy identified.

Subsequently, a left modified radical mastectomy was performed on October 21, 2024. During surgery, tissue in the superior axilla appeared indurated and inflamed, attributed to prior SLNB. Dissection included levels I and II of the axilla. The thoracodorsal and long thoracic nerves were preserved. Two drains were placed intraoperatively and removed on postoperative days 10 and 16. Final pathology revealed residual invasive lobular carcinoma measuring 17 mm in greatest dimension (Figure 5), with negative margins. Angiolymphatic invasion was present. Among the axillary contents, a hard, nodular lesion was identified. Pathologic evaluation showed chondroid and osseous metaplastic bone formation within granulation tissue (Figure 6). Immunostains were negative for pancytokeratin, Cytokeratin 7 (CK7), and p63, and the Ki-67 index was low, consistent with a benign reactive process rather than malignancy. The final pathologic stage was ypT1c, N2a.

Radiation therapy was initiated following surgical recovery. The patient received a total dose of 5000 centigray (cGy) in 25 fractions to the left chest wall using 10 megavolt (MV) photons and a field-in-field technique to optimize dose homogeneity. Concurrent radiation to the left supraclavicular region was administered through a right anterior oblique portal, also delivering 5000 cGy in 25 fractions. A posterior axillary boost was delivered via a posterior portal to a dose of 575 cGy in 25 fractions. Surveillance computed tomography (CT) chest imaging in November 2024 showed stability of the right lung nodule. Diagnostic mammography of the right breast in April 2025 showed no suspicious findings.

In January 2025, the patient was started on monthly leuprolide injections and oral exemestane for endocrine therapy. Additionally, she began treatment with abemaciclib as part of an adjuvant regimen for hormone receptor-positive, node-positive disease. She remains in follow-up with no evidence of recurrence or metastatic progression. Plans for delayed reconstruction are under evaluation.

## Discussion

ILC presents a unique clinical and pathological profile that differentiates it from other breast cancer subtypes [5]. Characterized by its loss of E-cadherin, ILC exhibits a discohesive, single-file infiltration pattern that frequently eludes early detection by physical examination or standard mammographic screening [16]. While typically hormone receptor-positive and HER2-negative, ILC is often diagnosed at a more advanced stage due to its subtle imaging appearance and diffuse growth [3]. This subtype also tends to show higher rates of multifocal and multicentric involvement, which may further complicate staging and surgical planning [16]. In this context, MRI has emerged as a valuable tool for assessing disease extent and guiding initial management [4].

The role of neoadjuvant chemotherapy in hormone receptor-positive ILC remains nuanced [10]. While patients with high-risk clinical features such as nodal involvement or high Ki-67 index may be appropriate candidates for chemotherapy, response rates in ILC are often less robust compared to invasive ductal carcinoma [17]. Nonetheless, neoadjuvant treatment can offer the opportunity to assess tumor biology in vivo and tailor surgical management accordingly [17]. In cases where axillary nodes are known to be involved before treatment, pre-treatment clip placement and post-treatment localization strategies, such as the use of Savi Scout markers, allow for targeted resection during surgery [11]. This approach supports accurate staging while potentially avoiding unnecessary dissection.

Sentinel lymph node biopsy following neoadjuvant chemotherapy introduces specific challenges, particularly when lymphatic drainage patterns have been disrupted by treatment [3]. In this patient, no hot or blue nodes were identified despite dual tracer use, which required excision of the previously clipped and palpable nodes instead. This situation highlights the need for careful coordination among the oncology, radiology, and surgical teams when planning axillary management before systemic therapy begins. While sentinel node mapping remains a widely accepted approach, some patients will still require completion axillary dissection if there is residual nodal disease or if localization proves unsuccessful [8].

A distinctive aspect of this case was the identification of benign chondroid and osseous metaplasia within axillary tissue. Heterotopic ossification in breast cancer surgery is exceptionally rare and can be mistaken for persistent or recurrent disease, especially when encountered as a firm, palpable mass [18]. The development of such a reaction is thought to be related to local trauma, chronic inflammation, or previous biopsy, which may stimulate pluripotent stromal cells to undergo mesenchymal differentiation [18]. This phenomenon has been documented in association with radiation, trauma, and even idiopathic cases but remains largely underrecognized. Intraoperatively, these lesions may resemble involved lymph nodes, raising concern for incomplete resection or treatment failure [14].

Histologic confirmation is crucial to distinguishing benign metaplastic processes from malignancy [19]. The absence of cytologic atypia and negative immunostaining for epithelial markers such as pancytokeratin, CK7, and p63 support a non-neoplastic diagnosis [17]. This distinction is essential to avoid over-staging, over-treatment, or unwarranted escalation of systemic therapy. In this patient, the metaplastic lesion was interpreted as a reactive change likely resulting from previous biopsy and chemotherapy-related tissue remodeling. Its accurate classification spared the patient additional intervention and reinforces the value of a thorough pathology evaluation in complex surgical specimens.

The adjuvant treatment strategy implemented for this patient reflects current best practices for premenopausal women with node-positive, hormone receptor-positive breast cancer [10,13]. Ovarian suppression with leuprolide in combination with an aromatase inhibitor improves disease-free survival in this group, especially when paired with a CDK4/6 inhibitor such as abemaciclib [19]. This combination has shown benefit in reducing recurrence risk and is particularly relevant in patients with high-risk pathologic features, including multiple positive nodes and angiolymphatic invasion [20]. Multimodal therapy incorporating systemic treatment, surgery, and radiation offers the best chance for long-term disease control. Careful attention to unusual histologic findings during treatment planning can optimize outcomes and reduce the risk of unnecessary intervention.

## Conclusions

This case reinforces several key clinical and educational principles in the management of invasive lobular carcinoma. First, the subtle radiographic and histologic features of ILC necessitate a high index of suspicion and often require advanced imaging such as MRI for accurate staging. Second, while neoadjuvant chemotherapy may offer limited tumor shrinkage in hormone receptor-positive ILC, it remains important in guiding surgical planning, particularly in patients with nodal disease. Third, the absence of sentinel node tracer uptake following neoadjuvant therapy illustrates the limitations of lymphatic mapping in treated axillae and highlights the importance of pre-treatment localization of involved nodes. Most notably, the identification of a rare benign chondroid and osseous metaplastic reaction within axillary tissue serves as a reminder that not all palpable or radiologically abnormal findings represent malignancy. Accurate histopathologic evaluation is essential to differentiate such lesions from metastatic disease, ensuring appropriate staging and avoiding unnecessary treatment escalation.
